# One-Step Synthesis of Self-Stratification Core-Shell Latex for Antimicrobial Coating

**DOI:** 10.3390/molecules28062795

**Published:** 2023-03-20

**Authors:** Guanzhou Zhen, Yuanchun Mu, Peichen Yuan, Yankun Li, Xiaoyu Li

**Affiliations:** 1 Beijing Engineering Research Center of Synthesis and Application of Waterborne Polymer, Beijing University of Chemical Technology, Beijing 100029, China; 2 State Key Laboratory of Organic-Inorganic Composite, Beijing University of Chemical Technology, Beijing 100029, China

**Keywords:** core-shell structure, quaternary ammonium salt, silicone acrylate emulsion, antibacterial property, antiviral property

## Abstract

Herein, we describe a one-step method for synthesizing cationic acrylate-based core-shell latex (CACS latex), which is used to prepare architectural coatings with excellent antimicrobial properties. Firstly, a polymerizable water-soluble quaternary ammonium salt (QAS-BN) was synthesized using 2-(Dimethylamine) ethyl methacrylate (DMAEMA) and benzyl bromide by the Hoffman alkylation reaction. Then QAS-BN, butyl acrylate (BA), methyl methacrylate (MMA), and vinyltriethoxysilane (VTES) as reactants and 2,2’-azobis(2-methylpropionamidine) dihydrochloride (AIBA) as a water-soluble initiator were used to synthesize the CACS latex. The effect of the QAS-BN dosage on the properties of the emulsion and latex film was systematically investigated. The TGA results showed that using QAS-BN reduced the latex film’s initial degradation temperature but improved its thermal stability. In the transmission electron microscopy (TEM) photographs, the self-stratification of latex particles with a high dosage of QAS-BN was observed, forming a core-shell structure of latex particles. The DSC, TGA, XPS, SEM, and performance tests confirmed the core-shell structure of the latex particles. The relationship between the formation of the core-shell structure and the content of QAS-BN was proved. The formation of the core-shell structure was due to the preferential reaction of water-soluble monomers in the aqueous phase, which led to the aggregation of hydrophilic groups, resulting in the formation of soft-core and hard-shell latex particles. However, the water resistance of the films formed by CACS latex was greatly reduced. We introduced a p-chloromethyl styrene and n-hexane diamine (p-CMS/EDA) crosslinking system, effectively improving the water resistance in this study. Finally, the antimicrobial coating was prepared with a CACS emulsion of 7 wt.% QAS-BN and 2 wt.% p-CMS/EDA. The antibacterial activity rates of this antimicrobial coating against *E. coli* and *S. aureus* were 99.99%. The antiviral activity rates against H_3_N_2_, HCoV-229E, and EV71 were 99.4%, 99.2%, and 97.9%, respectively. This study provides a novel idea for the morphological design of latex particles. A new architectural coating with broad-spectrum antimicrobial properties was obtained, which has important public health and safety applications.

## 1. Introduction

The continued prevalence of COVID-19 in recent years has seriously affected people’s daily lives, causing substantial economic losses and social problems worldwide. Transmission through contaminated surfaces is an important way of spreading microbial infections. By exploring surfaces and coatings that minimize the survival of disease-causing microorganisms and applying them to public places, we can effectively impede the spread of microorganisms and reduce the risk of disease infection [1,2]. Antimicrobial coatings are surface materials that reduce the adhesion of microorganisms (bacteria, fungi, and viruses) or kill microorganisms on their surface [3]. Three significant ideas are currently being developed for antimicrobial coatings. The first is a release-type antimicrobial strategy, adding antimicrobial agents such as antibiotics [4], halogens [5], and silver ions [6] to give the coating an antimicrobial effect. These bactericidal agents slowly disperse in the environment to inhibit microorganisms. The second method is to reduce the surface adhesion of microorganisms. This solution is achieved by increasing the hydrophobicity [7] or hydrophilicity [8] of the coating surfaces so that bacteria or viruses cannot bind to these surfaces or be easily washed off. The third type is a structural antimicrobial coating [9], which kills microorganisms by surface modification. Structural antimicrobial coatings are less prone to the loss of antimicrobial groups due to strong chemical bonding between the antimicrobial groups and the main resin. Therefore, they can maintain long-term effective antimicrobial performance without polluting the environment [10].

Quaternary ammonium salt (QAS) is a highly efficient, green, and long-lasting antimicrobial agent and has therefore received much attention [11,12,13]. A long-term effective antimicrobial material can be obtained by introducing QAS into the copolymer chain. However, due to the antimicrobial mechanism of QAS, it is difficult for QAS to be effective against non-enveloped viruses. Recent studies have shown the antibacterial mechanism of QAS: Firstly, they adsorb on the surface of negatively charged lipid cell membranes through positively charged quaternary ammonium groups. Then, they penetrate the cell membrane through long chains, leading to structural disruption and the efflux of intracellular material. Finally, microorganisms such as bacteria and fungi are killed [14]. Therefore, the current mainstream QAS antimicrobial scheme involves the disruption of the membrane structure through the long chain structure, and different forms of QAS antimicrobial agents, such as single-chain QAS [15], double-chain QAS [16], double QAS [17], and poly-QAS [18], have been developed. Since QAS achieves the inactivation of microorganisms by disrupting the membrane structure, QAS is equally capable of inactivating enveloped viruses, but it has difficulty inactivating non-enveloped viruses. However, some studies have shown that certain quaternary antimicrobial agents can be effective against envelope-free viruses. For example, Kawabata [19] found that crosslinked N-benzyl-4-vinylpyridinium bromide copolymer (BVP resin) reduced the infectivity levels of enterovirus, herpes zoster virus, poliovirus, and human immunodeficiency virus suspensions by a factor of 1000–100,000 within 2 h. In addition, Wood [20] found that benzalkonium chloride showed antiviral activity against envelope-free coxsackievirus and common human ocular adenovirus, and that the antiviral activity correlated with the concentration of benzalkonium chloride. The above study mentioned two antimicrobial materials capable of inactivating envelope-free viruses. Their common point was that they both possessed a benzyl group linked to an ionic group. Therefore, the QAS with benzylic properties had the potential to become a broader-spectrum antimicrobial material.

The distribution of quaternary ammonium groups in the particles influences the antimicrobial property of the coatings. With the help of the design of the core-shell structure of the latex particles, the effective groups are concentrated in the shell layer of the latex particles, thus giving better play to the antimicrobial property. The synthesis of latex particles with a core-shell morphology is an area of prevalent research that has been receiving extensive attention [21,22,23,24]. Due to ionic bonding [25], grafting, or interpenetrating networks [26] between the core and the shell layer, particles with a core-shell structure can improve the impact resistance, abrasion resistance [27], water resistance [28], damping properties [29], and a variety of other properties [30] of copolymers, unlike non-core-shell-structure copolymers and polymer blends [31]. Different preparation methods can produce latex particles with different structures from the same material. Changing the layer thickness of the core-shell structures or the contents of other materials in the layers can also produce many excellent properties, giving them a wide range of applications [30,32]. For example, in the field of biomedicine, core-shell nanoparticles are mainly used for controlled drug transport [33], the imaging of organisms, cellular labeling [34], biosensors [35], and regenerative medicine [36]. Through scientific design, it is possible to obtain core-shell and variously shaped structures, including strawberry-type [37], snowman-type, and island-type [38]. For emulsion polymerization, the dominant approach to designing core-shell latex particles is a multi-step reaction, which also allows the formation of a multilayer core-shell structure. The one-step reaction generally results in homogeneous latex particles [39]. The core-shell latex particles obtained by the one-step reaction are generally organic–inorganic composites [40].

In this work, we used a one-step emulsion polymerization reaction to prepare the CACS latex particles for the antimicrobial coatings, which used 2,2’-azobis(2-methylpropionamidine) dihydrochloride (AIBA) as the water-soluble initiator and butyl acrylate (BA), methyl methacrylate (MMA), vinyltriethoxysilanec (VTES), water-soluble polymerizable QAS-BN (synthesized from 2-(Dimethylamine) ethyl methacrylate, and benzyl bromide) as reactants. QAS-BN was first synthesized, and ^1^H NMR and FTIR demonstrated the structure of the monomer. The well-defined core-shell latex particles were observed under transmission electron microscopy (TEM) as the amount of QAS-BN used was increased. The DSC, DMA, TEM, etc., also demonstrated the core-shell structure of the latex particles. The introduction of QAS-BN improved the stability of the CACS emulsion and provided broad-spectrum antimicrobial properties against bacteria, molds, and viruses. However, the introduction of QAS-BN also led to a decrease in the water resistance of the latex films. We improved the water resistance of the latex films using a low-temperature crosslinking system formed by p-chloromethyl styrene and n-hexane diamine (p-CMS/EDA). The result was a broad-spectrum antimicrobial coating that could be used in hospitals, shopping malls, schools, and other public places to reduce the transmission of diseases.

## 2. Results

### 2.1. Copolymer Verification

**Figure 1:**
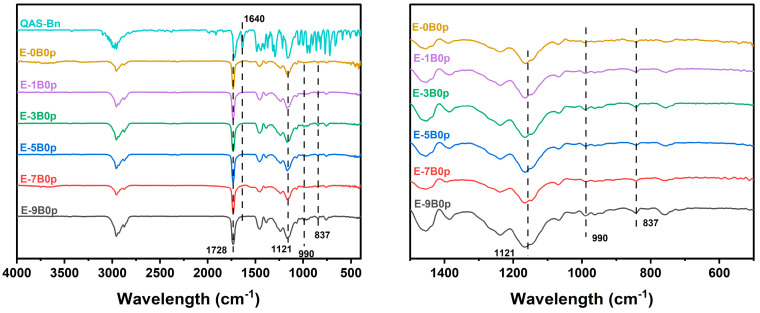
The FTIR spectra of acrylate emulsions and the monomer QAS-BN.

Cationic acrylate emulsions with different contents of QAS-BN were prepared. The FTIR spectra of the cationic acrylate emulsions and monomer QAS-BN are presented in Figure 1. Within them, the 1640 cm^−1^ position is the stretching peak of the methacrylic acid double bond, which disappeared from the FTIR spectrum of the polymer emulsion sample, indicating that the monomer had completed the copolymerization reaction and there was no unreacted monomer in the emulsion samples. The 1728 cm^−1^ position is the stretching vibration peak of the carbonyl group, indicating the successful copolymerization of BA and MMA to the copolymer molecular chain. The 990 cm^−1^ position is the characteristic peak of the quaternary ammonium group, meaning that QAS-BN had been successfully polymerized into the copolymer molecular chain. The broad peak at 1121 cm^−1^ is the antisymmetric stretching vibration peak of Si-O-Si. The characteristic absorption peak of Si-C at 837 cm^−1^ indicated that the silicone had been successfully copolymerized to the polymer chains. The above characteristic peaks were reflected in the IR patterns of these emulsion samples with different QAS-BN dosages, indicating that the copolymerization of the reactive monomers BA, MMA, VTES, and QAS-BN was completed for all the emulsion samples.

### 2.2. The Effect of QAS-BN Dosage on the Emulsion Performance

The test results of the emulsion samples with different QAS-BN dosages are shown in Table 1. Generally, the cationic acrylate emulsions with varying contents of QAS-BN had a milky blue appearance, and the emulsions could be stored for a long time without precipitation and separation. When the content of QAS-BN did not exceed 3 wt.%, the conversion rate of the emulsion was high (>99.8%); however, when the content of QAS-BN exceeded 3 wt.%, the conversion rate gradually decreased. For example, the conversion rates were 98.60%, 96.45%, and 91.63% when the content of QAS-BN was 5 wt.%, 7 wt.%, and 9 wt.%, respectively. Additionally, gels of 1.21% and 5.32% occurred in the samples with QAS-BN dosages of 7 wt.% and 9 wt.%, respectively. This indicated that the stability of the emulsion decreased when the content of QAS-BN was greater than 7 wt.%. The decrease in emulsion stability may have been due to the production of water-soluble oligomers, which increased with the amount of QAS-BN. The presence of oligomers in water rich in ions would increase the ionic strength and thus decrease the stability at a high content, including inducing coagulation [41].

#### 2.2.1. Size and Zeta Potential of Latex Particles

**Figure 2:**
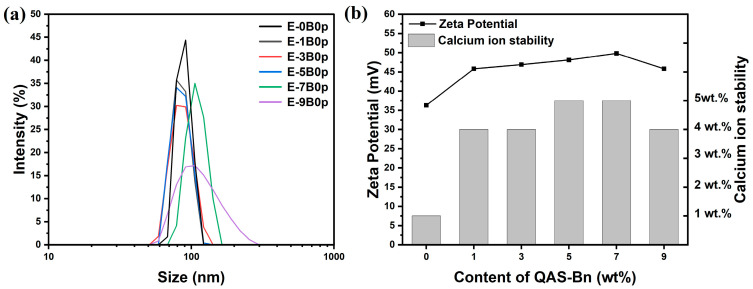
(**a**) Particle size of emulsions with different QAS-BN dosages; (**b**) zeta potential and calcium ion stability of emulsions with different QAS-BN dosages.

Table 1 and Figure 2 show that the particle size and distribution of the particles increased with the rise in QAS-BN dosage. Still, when the dosage of QAS-BN was less than 3 wt.%, the growth of the emulsion was small, and the particle size only increased from 82.61 nm to 83.95 nm. However, when the dosage of QAS-BN was increased to 5 wt.%, the growth of the emulsion particle size increased significantly (from 83.95 nm to 88.06 nm). The content of QAS-BN changed from 5 wt.% to 9 wt.%, the particle size increased from 88.06 nm to 115.4 nm, and the PDI increased from 0.066 to 0.171. The increase in latex particle size with the rise in QAS-BN dosage was due to the strong hydrophilic ability of QAS-BN as an ionic monomer. Adding it to the system caused the molecular chains to tend to extend in the aqueous phase, thus increasing the particle size. When the content of QAS-BN exceeded 5 wt.%, the increase in particle size was due to the morphological changes of the latex particles, which will be explained later in conjunction with the morphology observed using TEM. 

**Figure 3:**
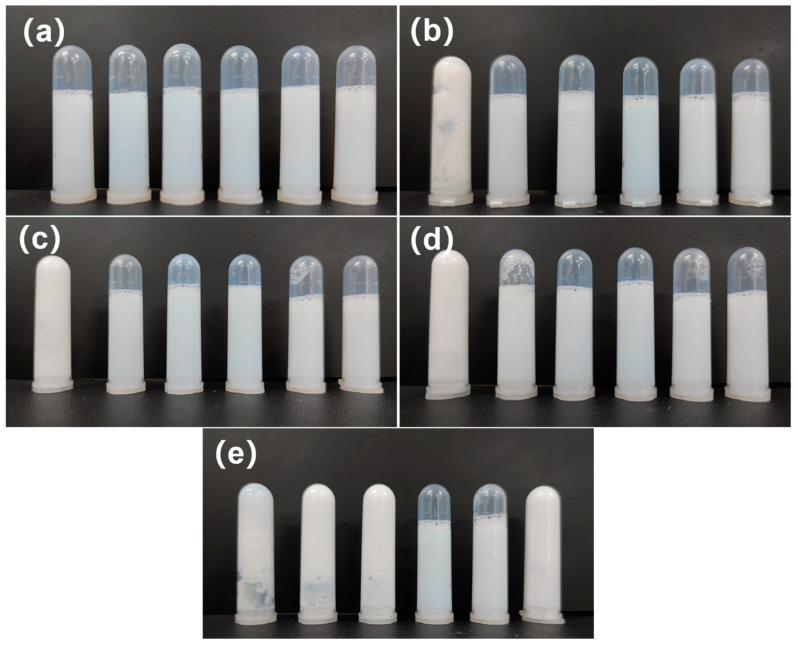
Calcium ion stability test results: (**a**–**e**) the mass fractions of calcium chloride solution used were 1 wt.%~5 wt.%, and the test samples are E-0B0p, E-1B0p, E-3B0p, E-5B0p, E-7B0p, and E-9B0p from left to right.

According to the zeta potential of the emulsion, it was found that the synthesized particles all had positive potential, indicating that they were cationic emulsions. When the QAS-BN dosage was 0 wt.%, the zeta potential of the emulsion was +36.3 mV, provided by the cationic emulsifier. As the QAS-BN dosage increased from 1 wt.% to 7 wt.%, the zeta potential of the emulsion showed an increasing trend, which indicated that the use of cationic monomers would improve the stability of the emulsions. However, when the content of QAS-BN was 9 wt.%, the zeta potential of the emulsion decreased to +45.8 mV. Combined with the fact that the conversion of the emulsion was only 91.63% at this time, 5.35 wt.% gels appeared during the emulsion synthesis, indicating that the emulsion system became unstable at this time. The zeta potential of the emulsion directly affected the calcium ion stability of the emulsion. The calcium ion stability is an important criterion to judge whether an emulsion can be used in architectural coatings. Because the preparation of architectural coatings requires the addition of calcium carbonate and other components of fillers, emulsions with poor stability will break during the configuration process. Table 1 shows that the calcium ion stability of E-0B0p, E-1B0p, E-3B0p, E-5B0p, E-7B0p, and E-9B0p was 1 wt.%, 4 wt.%, 4 wt.%, 5 wt.%, 5 wt.%, and 4 wt.%, respectively. The emulsion states after mixing the samples and different concentrations of calcium chloride solutions are shown in Figure 3. The test results for calcium ion stability and the emulsion zeta potential were highly consistent. These experiments further supported the effect of QAS-BN on the emulsion’s stability.

#### 2.2.2. Morphology of Latex Particles

**Figure 4:**
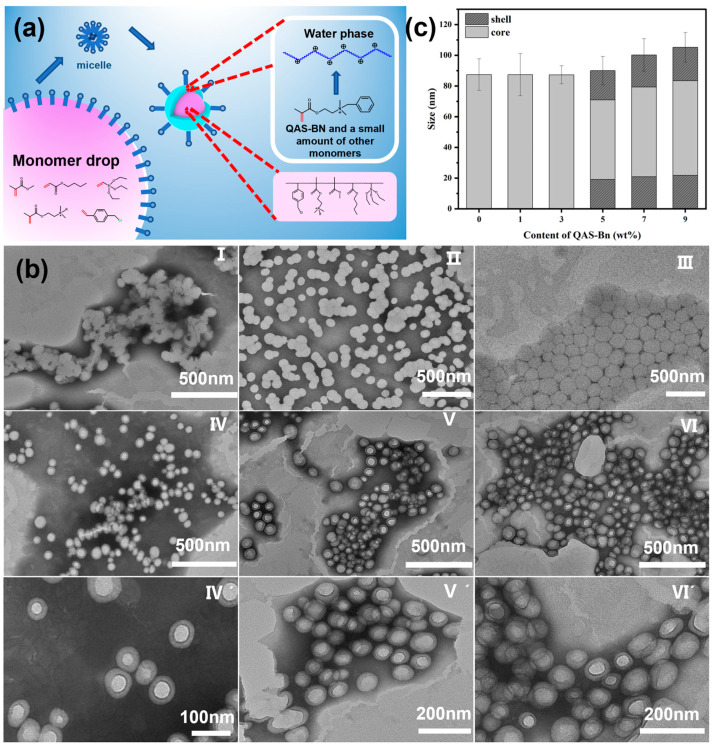
(**a**) Schematic diagram of the formation process of CACS emulsion particles; (**b**) TEM of latex with different QAS-BN dosages (I—E-0B0p; II;—E-1B0p; III—E-3B0p; IV/IV’—E-5B0p; V/V’—E-7B0p; VI/VI’—E-9B0p); (**c**) average size of latex particles calculated from TEM images.

The self-stratification process of latex particles by the one-step method is shown in Figure 4a. 

Because the water-soluble initiator AIBA was used, the reactive radicals of the reaction were first formed in the aqueous phase. The initial reactive radicals were obtained from small amounts of monomers dissolved in water for acrylate emulsions using conventional water-soluble initiators. For example, the solubility of MMA in water is 1.59 wt.% (20 °C). Since the quaternary ammonium monomer used in this study is completely soluble in the aqueous phase, a large percentage of the monomer QAS-BN was dissolved in the aqueous phase at the beginning of the reaction. Additionally, the homopolymer of QAS-BN is soluble in water. Therefore, when an aqueous emulsion polymerization reaction with a water-soluble initiator and water-soluble monomers is carried out, it proceeds through the following stages: (a) the initiator and monomers in the aqueous phase combine to produce reactive radicals; (b) the monomers in the aqueous phase undergo solution polymerization due to the presence of water-soluble monomers to produce water-soluble oligomers [41,42]; (c) the molecular chain grows to a certain level and then enters the micelle to participate in the reaction due to the decrease in water solubility. Due to the above process, the quaternary ammonium groups were concentrated on certain molecular chains. During the reactions taking place in the micelles, the hydrophilic groups brought by QAS-BN caused the molecular chains to move toward the peripheral aqueous phase, and the latex particles spontaneously stratified, forming a shell structure with a higher percentage of QAS-BN.

The structures of latex particles with different QAS-BN dosages were observed by TEM, as shown in Figure 4b. The cationic acrylate particles with different QAS-BN dosages had a stable spherical structure. Specifically, the latex particles of the E-0B0p, E-1B0p, and E-3B0p samples did not show phase separation or core-shell structures. However, a significant particle coalescence of sample E-0B0p could be observed; the latex particles of E-1B0p also showed particle coalescence, but it was not as apparent as that of E-0B0p, while E-3B0p showed the deformation of the adjacent latex particles but did not coalesce. This indicated that the coalescence ability of the latex particles gradually decreased with the increase in QAS-BN dosage. The latex particles of the other samples, E-5B0p, E-7B0p, and E-9B0p, showed two clear lining regions under TEM: a lighter region in the center, and a darker region towards the outside. This suggested that the synthesized latex particles had a distinct two-phase structure, typical of core-shell particles. This implied that the phase separation of the latex particles with a core-shell structure occurred spontaneously during the copolymerization. Moreover, the latex particles of the E-5B0p, E-7B0p, and E-9B0p samples were stacked together, but the boundaries between the latex particles were obvious, and almost no deformation or coalescence occurred. This suggested that the shell layer of the CACS latex particles may have been composed of a hard phase with a high Tg. Therefore, CACS latex particles with a soft core and hard shell were formed. The harder shell layer prevented the coalescence between the latex particles, which explained the observation that the latex particles with a core-shell structure still had clear boundaries despite the accumulation. In contrast, the coalescence of the latex particles of E-0B0p, E-1B0p, and E-3B0p gradually decreased with an increasing QAS-BN dosage, indicating that although no core-shell structure was observed, the migration of hydrophilic hard segment monomers to the outer layer was also present. Still, no significant phase separation occurred due to the low dosage of QAS-BN.

We determined the size of the latex particles observed using TEM, and the statistical results are shown in Table 2. These results coincided with the particle size data obtained by the dynamic light-scattering particle size analyzer (DLS). The average particle sizes of the latex particles with different QAS-BN dosages were 82.61 nm, 115.4 nm, 82.97 nm, 83.95 nm, 88.06 nm, and 111.2 nm for the E-0B0p, E-1B0p, E-3B0p, E-55B0p, E-7B0p, and E-9B0p samples, which agreed with the CACS latex TEM micrograph measurements of 87.44 nm, 87.35 nm, 87.28 nm, 89.99 nm, 100.22 nm, and 105.20 nm, respectively. The particle size distribution index (PDI) values were 0.017, 0.033, 0.040, 0.066, 0.118, and 0.171 for the corresponding CACS emulsion particles. When the QAS-BN dosage was less than 5 wt.%, the latex particle sizes did not change significantly, and both were about 87 nm. However, when the QAS-BN dosage exceeded 5 wt.%, the latex particle sizes increased significantly, from 89.99 nm to 105.20 nm. The important information obtained from the statistical results was the change in the thickness of the shell layer and the volume ratio. We could see that the core sizes of the latex particles of the E-5B0p, E-7B0p, and E-9B0p samples were 51.81 nm, 58.44 nm, and 61.69 nm, respectively, and the thicknesses of the shell layers were 19.09 nm, 20.89 nm, and 21.75 nm, respectively. The volume ratio of the shell layer of these CACS particles could be calculated from the statistical data, and the results were 66.85 *v*/*v*.%, 66.00 *v*/*v*.%, and 65.62 *v*/*v*.%. The volume ratio of the shell layer was much larger than the content of QAS-BN, which further indicated that the shell layer was not composed of homopolymers of QAS-BN. This was partly because QAS-BN was not the only monomer in the aqueous phase. Nevertheless, due to the larger solubility of QAS-BN in the aqueous phase, the reaction initially formed a larger proportion of QAS-BN in the molecular chain in the aqueous phase. Then, the molecular chains entered the micelles and continued to copolymerize with the main reaction monomer BA/MMA. The copolymer chain segments with denser quaternary ammonium groups pulled the copolymer molecular chains toward the aqueous phase in the initial reaction, thus spontaneously causing phase separation and producing a core-shell structure. This structure allowed the quaternary ammonium groups with an antimicrobial effect to be concentrated on the surface of the latex particles, which had a positive impact on the antimicrobial properties of the coating in the application process, as shown later.

#### 2.2.3. Thermal Properties of Latex Films

**Figure 5:**
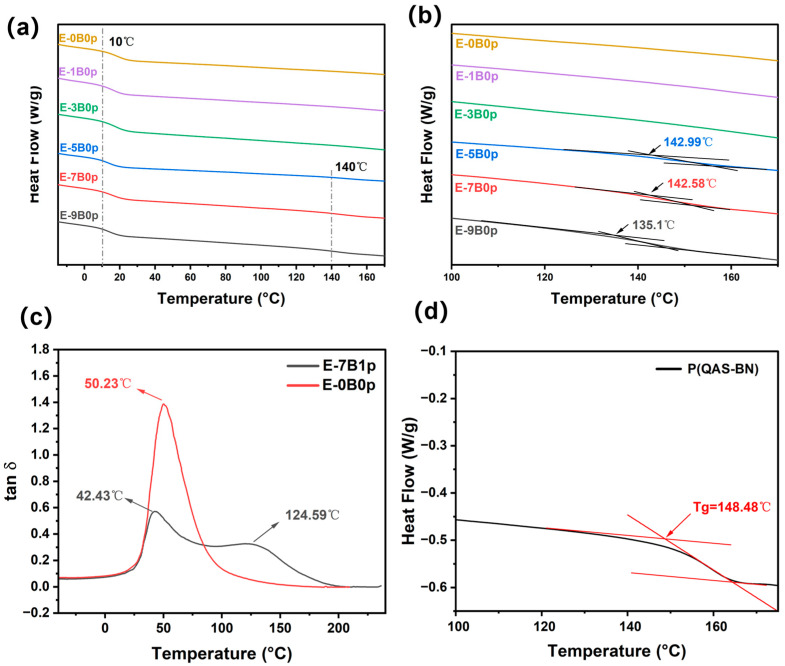
(**a**,**b**) DSC of latex films with different QAS-BN dosages. (**c**) DMA of latex films with different QAS-BN dosages. (**d**) DSC of QAS-BN homopolymer.

The DSC curve is shown in Figure 5a. It shows that all samples exhibited a similar Tg around 10 °C. Three samples, E-5B0p, E-7B0p, and E-9B0p, also had another Tg around 140 °C. The Tg values of samples E-5B0p, E-7B0p, and E-9B0p in the high temperature region were 142.99 °C, 142.58 °C, and 135.1 °C, respectively, as seen in Figure 5b. This indicated the existence of phase separation in the latex films, which was consistent with the core-shell structure observed under TEM. Sample E-0B0p (without a core-shell structure) and sample E-7B1p (with a core-shell structure) were further tested by DMA. The test results are shown in Figure 5c. Regarding the curve of the tan δ of the copolymer latex films according to the temperature, the curve of sample E-0B0p showed a peak at 50.23 °C, and the curve of sample E-7B1p showed two peaks at 42.43 °C and 125.90 °C. This indicated no phase separation in the latex film when the QAS-BN addition was 0 wt.%. Nevertheless, there was a significant phase separation when the addition was 7 wt.%, which was caused by the core-shell morphology of the latex particles, where 42.43 °C was the Tg of the core layer, and 124.59 °C was the Tg of the shell layer. This was consistent with the results of the latex particle morphology observed by TEM. Normally, the Tg values obtained by DSC and DMA are not the same, which is caused by the different testing methods. The FOX equation yielded a Tg of about 3.53 °C when the BA/MMA ratio was 1:1. This indicated that the core layer of the latex particles was mainly a BA/MMA copolymer; the Tg of the copolymer of QAS-BN was 148.48 °C, as shown in Figure 5d. The Tg of the shell layer of the latex particles was slightly lower than 148.48 °C, indicating that the shell layer of the latex particles was mainly composed of copolymers of QAS-BN and a small amount of the remaining monomers. The lower Tg of the shell layer of sample E-9B0p compared to E-7B0p and E-5B0p was caused by the loss of QAS-BN due to gelation during the emulsion polymerization. It was further demonstrated that in the emulsion polymerization reaction using a water-soluble initiator, when the water-soluble monomer reached a certain concentration in the aqueous phase, latex particles were formed that spontaneously delaminated to generate a core-shell structure of latex particles.

**Figure 6:**
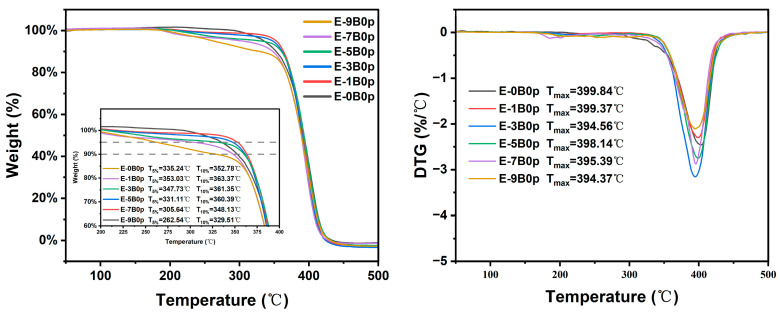
TGA results of latex films with different QAS-BN dosages.

The TGA results are shown in Figure 6 and Table 3. The T_5%_ (5% mass loss) was used to represent the onset temperature of degradation, and the T_10%_ (10% mass loss) was used to evaluate the thermal stability of the latex films. The T_5%_ of samples E-0B0p, E-1B0p, E-3B0p, E-5B0p, E-7B0p, and E-9B0p was 335.24 °C, 353.03 °C, 347.73 °C, 331.11 °C, 305.64 °C, and 262.54 °C, respectively; the T_10%_ was 352.78 °C, 363.37 °C, 361.35 °C, 360.39 °C, 348.13 °C, and 329.51 °C, respectively. Except for sample E-0B0p, the T_5%_ and T_10%_ values of the samples gradually decreased with the increase in QAS-BN dosage. This indicated that the thermal stability of the latex films gradually decreased with the increase in QAS-BN dosage. This was caused by the decomposition of the quaternary ammonium group due to the lower bonding strength of the C-N bond than the C-C bond. However, the T_5%_ and T_10%_ values of sample E-0B0p were lower than those with 1 wt.% and 3 wt.% QAS-BN added. The observation of the curves revealed that the weight loss of E-0B0p was low until the temperature was increased to 250 °C. This indicated that introducing QAS lowered the latex film’s initial decomposition temperature but improved the polymer’s thermal stability [36].

#### 2.2.4. XPS of Latex Film

**Figure 7:**
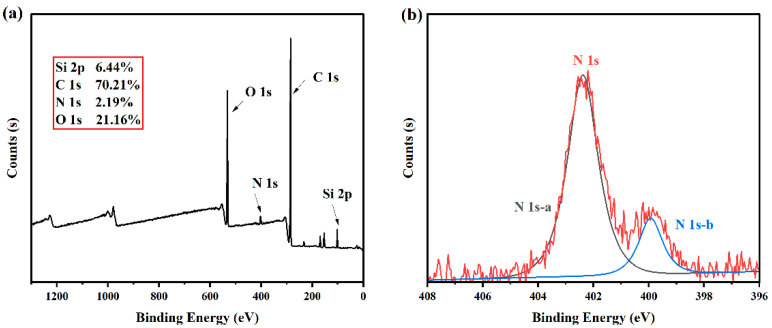
(**a**) XPS spectral results of sample E-7B0p; (**b**) N-element peaks in XPS spectral results of sample E-7B0p.

Figure 7a shows that XPS spectral results revealed the characteristic signals of C, O, N, and Si elements in sample E-7B0p. We could see that the percentage of nitrogen atoms on the surface of the copolymer film was 2.19%, which was higher than the theoretical value (0.55%), indicating that the quaternary ammonium groups were enriched on the surface of the copolymer film prepared by the CACS emulsion with a one-step polymerization method in this study. In addition, as shown in Figure 7b, two N existed in two states on the surface of the latex film, with binding energies of 402.38 eV and 399.93 eV, respectively. The peak area ratio was about 5:1. According to the analysis, the N at 402.38 eV existed as R_4_N^+^. In contrast, that at 399.93 eV existed in the form of R_3_N. This suggested that some of the quaternary ammonium groups degraded to tertiary amines during the reaction or film formation process due to the poor heat resistance of QAS. However, the results of the subsequent antimicrobial experiments showed that this did not have a significant effect.

#### 2.2.5. Surface Morphology of Latex Film

**Figure 8:**
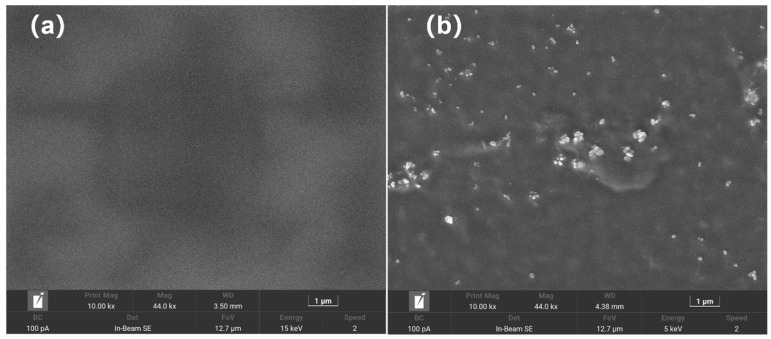
(**a**,**b**) Surface morphology of latex film observed using SEM (**a**—E-1B0p; **b**—E-7B0p).

The surface morphology of the latex films observed by SEM are shown in Figure 8. The surface of the latex film of the E-1B0p sample had a uniform structure and smooth surface. The surface of the latex film of the E-7B0p sample featured protruding particles, which were fused latex particles without complete melting. This was because the latex particles of the E-0B0 sample were soft-core and hard-shell latex particles. The latex film’s surface morphology also corroborated the latex particles’ morphological results.

#### 2.2.6. Hydrophobicity of Latex Film

**Figure 9:**
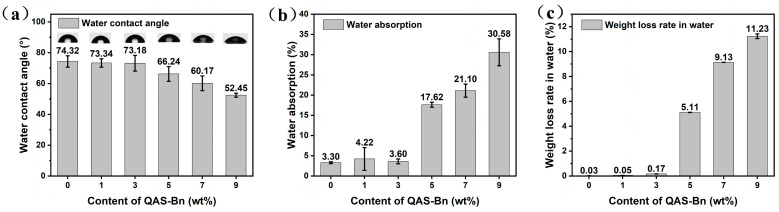
(**a**–**c**) Water contact angle, water absorption rate, and weight loss rate in the water of the latex films with different QAS-BN dosages.

When the content of QAS-BN was increased from 0 wt.% to 9 wt.%, the water contact angle and the absorption rate of the latex films were tested, as well as the weight loss of the latex films when immersed in water for 7 day. The test results are shown in Figure 9, demonstrating that the changes in these three indicators were strongly correlated with the morphological changes of the latex particles presented in Figure 9b. When the contents of QAS-BN were 0 wt.%, 1 wt.%, and 3 wt.%, the contact angle of the latex film remained around 74°, and there was no significant decrease with the increase in hydrophilic monomers. Similarly, the water absorption of the latex film remained at about 4 wt.%, and there was no significant increase with the rise in QAS-BN dosage; at this point, the weight loss rate of the latex film in water was always close to zero, indicating that the mass loss due to the emulsifier was negligible. Combined with the morphology observed by TEM, we determined that when the content of QAS-BN was less than 3 wt.%, there was no significant phase separation in the latex particles. At this point, the hydrophilic groups tended to be uniformly dispersed in the latex particles, which was the reason for the above results. 

When the contents of QAS-BN reached 5 wt.%, 7 wt.%, and 9 wt.%, the water contact angle of the latex film was 66.24° ± 2.37°, 60.17° ± 2.37°, and 52.45° ± 0.65°, respectively, decreasing significantly with the increase in QAS-BN; the water absorption was 17.62% ± 0.30%, 21.10% ± 0.80%, and 30.58% ± 1.66%, respectively, decreasing significantly with the increase in QAS-BN; and the weight loss rate of the latex film in water was 5.11% ± 0.01%, 9.13% ± 0.01%, and 11.23% ± 0.10% respectively, also increasing significantly, but even higher than the total content of hydrophilic monomer added. When the QAS -BN dosage content increased from 3 wt.% to 5 wt.%, the water contact angle reduced from 73.18° ± 2.54° to 66.24° ± 2.37°; the water absorption rate directly increased from 3.60% ± 0.30% to 17.62 % ± 0.30%; and the weight loss rate in water reached 11.23% ± 0.01% from 0.17% ± 0.01%. The water resistance of the polymer latex film showed significant changes, which had a certain relationship with the morphologies of the latex particles. As mentioned above, when the content of QAS-BN exceeded 3 wt.%, the latex particles spontaneously formed a less hydrophilic core layer and a more hydrophilic shell layer. The enrichment of hydrophilic groups on the surface of the latex particles reduced the water resistance of the latex films. Even some of the more hydrophilic copolymer chain segments were dissolved by water, which led to the weight loss of the latex film. This could have a negative impact, because the loss of hydrophilic molecular chains directly affected the antimicrobial properties of the coating, so we used crosslinkers to improve the water resistance in the subsequent tests.

### 2.3. Effect of Crosslinking Agent

QAS-BN, as an antimicrobial functional monomer, needed to be added in as high a quantity as possible. However, since the emulsion became unstable when the content of QAS-BN increased to 9 wt.% and a large amount of gel was generated during the reaction, we chose to set the addition of QAS-BN at 7 wt.%. From the above examination of the effect of the QAS-BN dosage on the water resistance of the latex films, we found that the water resistance of the latex films without crosslinkers was poor, and even the antimicrobial effect produced by the structure was lost when immersed in water. The crosslinked structure of the films could improve their performance. DAAM and ADH are crosslinked systems commonly used in acrylate-based emulsions, but they have many problems, such as a sharp viscosity increase and poor stability. Therefore, this crosslinked system was unsuitable for the CACS emulsion in our lab. Thus, at this stage, p-CMS was introduced into the polymerization reaction in the experimental protocol. EDA aqueous solution was added after the completion of the reaction at room temperature to form a room-temperature crosslinking system. The effect of the p-CMS/EDA crosslinking system on the CACS emulsion and film properties was investigated.

The content of p-CMS/EDA was varied to prepare CACS emulsions, and the results of the performances are shown in Table 4. The particle size gradually increased from 111.2 nm to 133.8 nm, and the particle size distribution gradually expanded from 0.118 to 0.207 as the content of p-CMS increased from 0 wt.% to 3 wt.%. This was due to the strong hydrophilic monomer of p-CMS, which increased the hydrophilicity of the polymer chain segments after addition.

**Figure 10:**
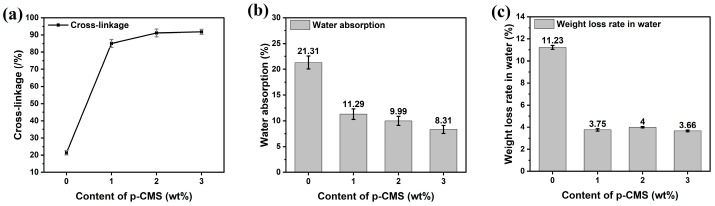
(**a**–**c**) Crosslinkage/water absorption/weight loss rate in the water of CACS emulsions with different p-CMS/EDA dosages.

The crosslinking degree of the latex films was determined by Soxhlet extraction, and the results are shown in Figure 10a. The crosslinking degrees of the E-7B0p, E-7B1p, E-7B2p, and E-7B3p samples were 21.36% ± 0.61%, 85.06% ± 1.61%, 91.11% ± 1.10%, and 91.76% ± 0.72%, respectively. This showed that adding 1 wt.% p-CMS/EDA could increase the crosslinking degree of the latex film from 21.36% ± 0.61% to 85.06% ± 1.61%, which indicated that the crosslinking system p-CMS/EDA could play a role in forming a crosslinking network. When the content of p-CMS/EDA was increased to 2 wt.%, the crosslinking degree of the latex film was further increased to 91.11% ±1.10%, after which the increase in the content of the crosslinking agent became smaller. The water resistance of the latex films was tested, and the results are shown in Figure 10b. The water absorption rates of the E-7B0p, E-7B1p, E-7B2p, and E-7B3p samples were 21.31% ± 0.63%, 11.29% ± 0.50%, 9.99% ± 0.43%, and 8.31% ± 0.40%, respectively, and the weight loss rates in water were 11.23% ± 0.10%, 2.75% ± 0.60%, 4.00% ± 0.03%, and 3.66% ± 0.04%. The results showed that after adding 1 wt.% p-CMS/EDA, the water resistance of the latex films was significantly improved. With a continued increase in the content of p-CMS/EDA, the water absorption of the latex films continued to decrease. Still, the change was not obvious, and the weight loss rate of the latex films in water was no longer significantly changed. This indicated that crosslinking agents could improve the prepared latex films’ water resistance.

**Figure 11:**
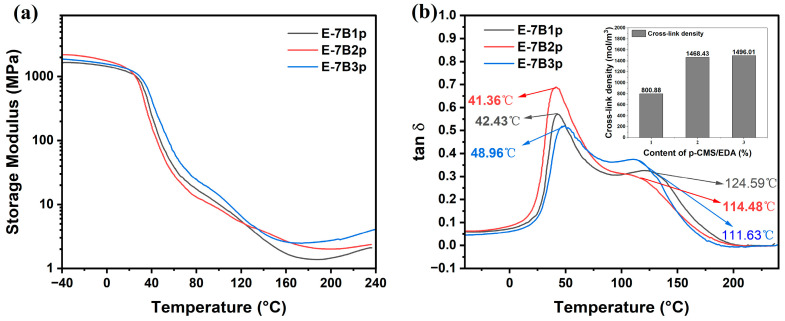
(**a**,**b**) The variation curves of storage modulus and tan δ with temperature for latex films with different contents of crosslinkers.

Figure 11 shows the DMA test results for samples with crosslinker additions from 1 wt.% to 3 wt.%. As a rule of thumb, the magnitude of the crosslinking density of a sample can be roughly estimated by the importance of the storage modulus in the platform region of the high-temperature region [43]. For example, Figure 11a shows that the storage modulus’s magnitude in the high-temperature zone’s platform region was E-7B3p > E-7B2p > E-7B1p. Therefore, the ranking of the magnitude of the crosslink density for these samples could be estimated as E-7B3p > E-7B2p > E-7B1p. In addition, according to the derivation equation of rubber elasticity theory, the following relationship exists between the crosslink density (V_E_) and the storage modulus (E) in the platform region of the rubber [44].

(1) $$V_{E_{1}}=\frac{E_{1}}{3\mathrm{RT}}$$

where E is the storage modulus in the rubber platform region above Tg in 0.1 Pa, usually measured at Tg + 40 °C; R is the common gas constant; and T is the absolute temperature.

As shown in Figure 11b, the Tg values of the latex films of the E-7B1p, E-7B2p, and E-7B3p samples were 42.43 °C, 41.36 °C, and 48.96 °C for the core layer and 124.59 °C, 114.48 °C, and 111.63 °C for the shell layer, respectively. The energy storage moduli of the samples beyond the shell layer Tg of 40 °C were 1.58902 MPa, 2.81856 MPa, and 2.89276 MPa, respectively. The above data are recorded in Table 5. The crosslink densities of the three samples E-7B1p, E-7B2p, and E-7B3p were calculated using Equation (1) as 800.88 mol/m^3^, 1468.43 mol/m^3^, and 1496.01 mol/m^3^, respectively. The results showed that the crosslinking density of the latex films rose gradually with the increase in the crosslinking agent dosage, but the amplification became insignificant over 2 wt.%. Therefore, according to the above results, we chose 2wt.% as the optimum amount of crosslinking agent.

### 2.4. Antimicrobial Properties of Coatings

**Figure 12:**
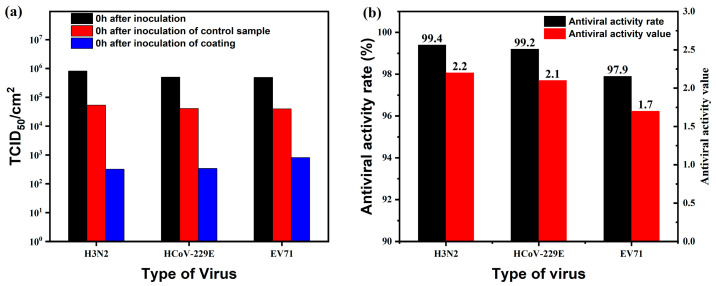
(**a**) Changes in the number of viruses after antiviral testing of the coating (E-7B2p); (**b**) Anti-viral activity value and anti-viral activity rate of the coating (E-7B2p).

**Figure 13:**
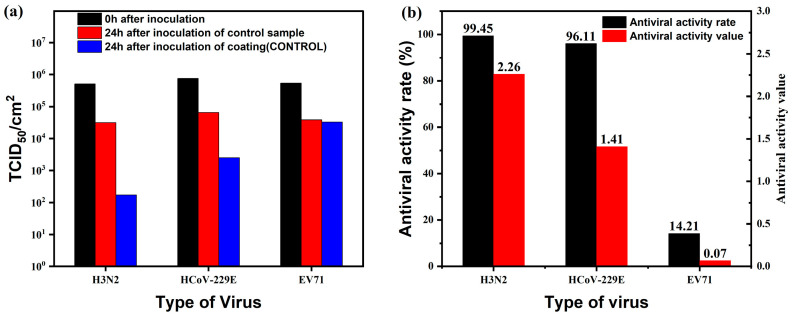
(**a**) Changes in the number of viruses after antiviral testing of the coating (control); (**b**) Anti-viral activity value and anti-viral activity rate of the coating (control).

After the above investigation, the coating (QAS-BN content 2.8 wt.%) was prepared using the E-7B2p emulsion. The prepared coating was applied uniformly on a 5 cm × 5 cm cement board to form a coating with a thickness of less than 100 um. The antimicrobial test of the coating was entrusted to Guangzhou Microbiological Analysis and Testing Center. The test report is provided in the supporting information. The antibacterial test results are shown in Table 6. The test results showed that this coating could completely kill *E. coli* and *S. aureus* after 24 h, and the antibacterial rate of the coating reached 99.99%. The coating could still completely kill the above bacteria after aging treatment, which indicated that the coating had a good anti-aging performance and lasting antibacterial performance. The constant-temperature and -humidity test method demonstrated the antimold performance of the coating. The test sample was placed in a glass cabinet for mold suspension spray drying for 10 min and then moved to the constant-temperature and -humidity room at 28 °C ± 1 °C, relative humidity 98% ± 1%. After 28 days, there was no mold growth observed under the microscope, indicating that the coating had excellent antimold performance. We conducted a preliminary study of the antiviral activity of the coating against influenza virus H_3_N_2_, coronavirus HCoV-229E, and enterovirus EV71. Figure 12 shows the changes in the number of active viruses after virus inoculation and the antiviral activity rate of the coating. The test results showed that the antiviral activity values of the coating against H_3_N_2_, HCoV-229E, and EV71 were 2.2, 2.1, and 1.7, respectively; the antiviral activity rates were 99.4%, 99.2%, and 97.9% after 24 h of virus inoculation. Figure 13 shows the change in the number of active viruses and the rate of antiviral activity of the coating from the control sample after virus inoculation. The antimicrobial functional structure of the control sample was a conventional long-chain-type quaternary ammonium salt. The test results showed that the antiviral activity values of the coating against H3N2, HCoV-229E, and EV71 after 24 hours of virus inoculation were 2.26, 1.41, and 0.07, respectively; the antiviral activity rates were 99.45%, 96.11%, and 14.21%. Both demonstrated an excellent inactivation ability against H3N2 and HCoV-229E, but the inactivation of HCoV-229E by the E-7B2p-coated sample was slightly better than that of the control sample. Both viruses were enveloped viruses. In contrast, the control sample showed almost no inactivation of the non-enveloped virus EV71, while the E-7B2p-coated sample could inactivate 97.9% of the EV71 virus after 24h. The prevailing view is that QAS cannot inactivate envelope-free viruses. This may be because the QAS with a benzyl functional group can inactivate the proteins of the virus shell. This suggests that QAS has a wider potential for broad-spectrum antimicrobial applications. The above results showed that the prepared coating had broad-spectrum antimicrobial properties and excellent resistance to bacteria, molds, and viruses, which are of great application value.

## 3. Materials and Methods

### 3.1. Materials

2-(Dimethylamine) ethyl methacrylate (DMAEMA), benzyl bromide, methyl methacrylate (MMA), butyl acrylate (BA), 2,2’-azobis(2-methylpropionamidine) dihydrochloride (AIBA), hexadecyl trimethyl ammonium bromide (CTAB), and hexyl hydride were all supplied by Macklin Reagent Co., Ltd. (Shanghai, China). Triethoxyvinylsilane (VTES) was purchased from Jinan Uno Chemical Co., Ltd. (Jinan, China). P-chloromethyl styrene (p-CMS) was purchased from Guangzhou Yuanda New Material Co., Ltd. (Guangzhou, China). All the above materials were analytical reagents used directly without treatment after purchase. The emulsifier, dispersant, defoamer, antifreeze, titanium dioxide, dicalcium, water-washed kaolin, calcined kaolin, film-forming additives, leveling agent, and thickening agent used in the coating preparation were all industrial-grade raw materials purchased from the market. All experimental water was deionized water.

### 3.2. Synthesis of Antimicrobial Functional Monomer

**Scheme 1:**
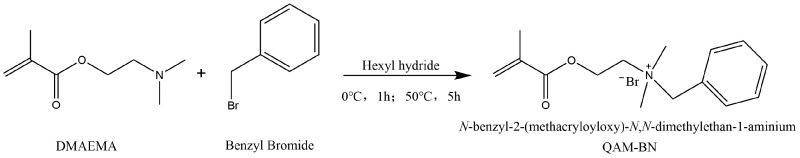
Reaction equation for the synthesis of antimicrobial functional monomers.

**Figure 14:**
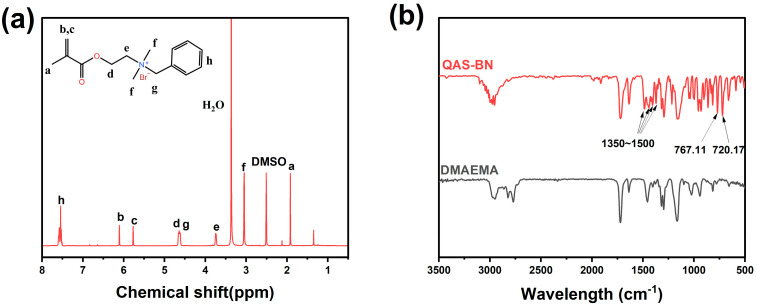
(**a**) ^1^H NMR spectrum of monomer QAS-BN; (**b**) FTIR spectra of monomer QAS-BN and reaction material DMAEMA.

The antimicrobial functional monomer was obtained by the Hoffman alkylation reaction [45,46,47] of benzyl bromide with DMAEMA. The reaction equation is provided in Scheme 1 (further synthesis mechanisms can be found in Scheme S1 from Supplementary Materials). Specifically, benzyl bromide and DMAEMA were mixed in hexyl hydride in a 1:1 ratio and stirred to react fully, and the solid pre-product was obtained by filtration. Next, the product was washed using hexane and acetone and dried under a vacuum to obtain a solid white product. The ^1^H NMR and FTIR spectra of the monomer are presented in Figure 14.

^1^H NMR (400 MHz, DMSO, 45 °C) δ(ppm): 7.54 (s, 5H, Ar); 6.10 (s, 1H, COCH_3_=CH_a_H); 5.77 (s, 1H, OCOCH_3_=CH_b_H); 4.64 (t, 2H, NCH+_2_); 4.64 (s, 2H, ArCH_2_); 3.75 (t, 2H, CO_2_CH_2_); 3.04 (s, 6H, NMe_2_); 1.92 (s, 3H, OCOCH_3_).

In the FTIR spectrum, the four peaks in the range of 1350–1500 cm^−1^ were bending vibrational peaks of the -CH=CH- of the benzene ring; the two peaks at 767.11 cm^−1^ and 720.17 cm^−1^ were out-of-plane bending peaks of the =CH of the monosubstituted benzene ring. This proved the successful substitution of benzyl for DMAEMA and indicated the successful synthesis of the monomer QAS-BN.

### 3.3. Synthesis of CACS Emulsion

**Figure 15:**
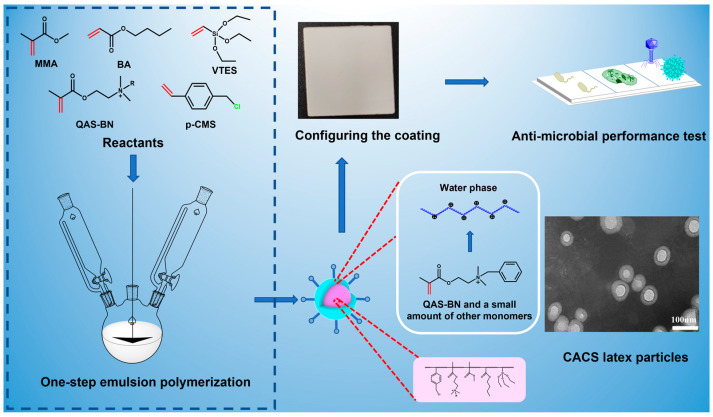
Experimental design flow chart.

CACS emulsions were prepared by the semi-continuous seed emulsion polymerization method, and the reaction flow is shown in Figure 15. Specifically, portions of the reaction monomers, emulsifiers, and water were mixed in a beaker, and the seed pre-emulsion was obtained by stirring with a magnetic stirrer. First, the seed pre-emulsion was added to a nitrogen-protected 1 L four-necked flask, stirred at 200 r/min, and raised to 87 °C in 20 min. Then, 30% of the total initiator was dissolved in water and added to this reaction flask. After 15 min of the reactants turning blue, the remaining reactant pre-emulsion and the aqueous solution of the initiator were added dropwise to the reaction flask within 120 min. After the addition was completed, the solution was maintained at 87 °C for 120 min. Finally, it was cooled to room temperature, supplemented with hexane diamine, and stirred for 30 min, and the emulsion was poured out. The emulsions with different contents of QAS-BN are listed in Table 7.

### 3.4. Coating Preparation

We made our colorant according to the formulation in Table 8. The stirring speed was controlled at 2800 r/min. The liquid additives were first dispersed in the container and then dispersed for 30 min after all powders were added. The dispersed colorant was transferred to the basket grinder for 25 min and collected in a jar for use.

The coating preparation was carried out according to the coating formulation in Table 9. One hundred grams of the CACS emulsion was placed in a beaker, and the film-forming auxiliary DPMA, colorant, and leveling agent BYK-333 were added and dispersed for 10 min at 1200 r/min for each step. After defoaming, a thickener was added to increase the viscosity. Finally, the coating was filtered with a 200-mesh filter, collected, and left for 24 h.

### 3.5. Characterization

#### 3.5.1. Emulsion Performance

Stability of calcium ions: The CaCl_2_ solutions (1 wt.%, 2 wt.%, 3 wt.%, 4 wt.%, and 5 wt.%) were configured separately, and 10 g of emulsion and 2 g of CaCl_2_ solution were mixed well and placed at room temperature for 48 h to observe the emulsion state. If the emulsion did not delaminate, break, or gel, the calcium ion stability test was passed.

Particle size and zeta potential: 1 mL of the emulsion was diluted to 500× and tested for particle size and distribution using a Zetasizer Nano ZS nanoparticle size analyzer.

Latex particle morphology characterization: After diluting the emulsion 40 times with deionized water and staining with 2 wt.% phosphotungstic acid solution, the morphology was characterized using a Hitachi HT7700 transmission electron microscope.

Solids content: We weighed the emulsion (*M*_1_), dried it to a constant weight, recorded this as *M*_2_, and calculated the solid content according to Formula (1).

(2) $$X=\frac{M_{2}}{M_{1}}\times 100\%\;$$

where *X* is the solid content of the emulsion sample (%), *M*_1_ is the sample mass before drying (g), and *M* is the sample mass after drying (g).

The conversion rate of the monomer was calculated by the dry weight method with the following formula:

(3) $$C=\frac{X}{X_{0}}\times 100\%\;$$

where *C* is the conversion rate of the monomer (%), *X* is the solid content of the emulsion sample (g), and *X*_0_ is the designed solid content of the emulsion sample (g).

#### 3.5.2. Film Performance 

Water contact angle: A film thickness of 100 μm was scraped onto a glass plate and dried at room temperature. The water contact angle of the sample film was measured using a video contact angle meter OCA20.

Water absorption and leaching rates: A square latex film (1 cm × 1 cm × 1 mm) was weighed and recorded as *M*_3_. The film was soaked in deionized water for 7d at room temperature, dried, and considered as *M*_4_. The film was dried, weighed, and recorded as *M*_5_. The water absorption rate (*A*) and weight loss rate in water (*L*) were calculated using the following formula:

(4) $$A=\frac{M_{4}-M_{3}}{M_{3}}\times 100\%\;$$

(5) $$L=\frac{M_{3}-M_{5}}{M_{3}}\times 100\%\;$$

Crosslinking degree: The extraction was carried out by the Soxhlet method with acetone as the solvent at reflux for 48 h, and the crosslinking degree was calculated according to Formula (5).

(6) $$C=\frac{M_{6}-M_{7}}{M_{6}}\times 100\%\;$$

where *C* is the crosslinking degree (%), $M_{6}$ is the initial weight of the latex film (g), and $M_{7}$ is the dried weight after the completion of Soxhlet extraction (g).

DSC analysis: A differential scanning calorimeter (DSC 1, Mettler Toledo Co., Ltd., Zurich, Switzerland) was used to test the latex film’s glass transition temperature (Tg). The heating rate was 10 °C/min, and the temperature range was −40 °C~150 °C.

DMA analysis: A dynamic mechanical analyzer (TAQ800-0604, TA Instruments Co., Ltd., Newcastle, DE, USA) was used to test and measure the latex films’ storage modulus and loss tangent (tan δ) values. The heating rate was 5 °C/min, and the temperature range was −40~240 °C.

XPS: An X-ray photoelectron spectrometer (ESCALAB 250) was used for the elemental analysis of the latex film’s surface.

SEM: Scanning electron microscopy (TESCAN MIRA LMS, Brno, Czech Republic) was used to characterize the surface morphology of the latex films.

#### 3.5.3. Antimicrobial Properties of Coatings

The durability performance test used the national standard specifications of a 30 W, 253.7 nm UV lamp with irradiation at a distance of 0.8 m–1.0 m from the test sample for 100 h.

Antibacterial performance and antibacterial durability experiments were performed according to GB/T 21866-2008, and the experimental strains were selected from *E. coli* and *Staphylococcus aureus*. Firstly, we prepared the bacterial solution at a concentration of (5.0~10.0) × 105 cfu/mL as the experimental solution and added 0.4 mL~0.5 mL of this solution to the negative control sample A, blank control sample B, and antibacterial coating sample C, respectively. The samples were covered with sterilized covering film by sterilized forceps and spread out to ensure no air bubbles. The bacteria evenly touched the samples, which were placed in sterilized dishes and incubated at (37 ± 1) °C and RH > 90% for 24 h. Three parallel tests were carried out for each sample. The samples were taken out and incubated for 24 h. Next, 20 mL of washing solution was added to sample A, sample B, sample C, and the covering film, and the samples were shaken well. The washing solution was inoculated into nutrient agar medium and incubated at (37 ± 1) °C for 24~48 h. The viable bacteria count in the washing solution was determined. We multiplied the above results by 1000 to obtain the actual recovered viable bacteria values of sample A, sample B, and sample C after 24 h incubation. The recovered bacteria value A of sample A should have been no less than 1.0 × 10^5^ cfu/tablet, and the actual recovered bacteria value B of sample B should have been no less than 1.0 × 10^4^ cfu/tablet. The three parallel viable values of the same blank control sample B needed to meet the condition that the highest logarithmic value of a minimum logarithmic value/the average logarithmic value of viable bacteria was less than or equal to 0.3.

The antibacterial rate was calculated by the following formula:

(7) $$R=\frac{B-C}{B}\times 100\%$$

where *R* is the antibacterial rate, *B* is the average number of recovered bacteria (cfu/tablet) after 24 h in the blank control plate, and *C* is the average number of bacteria recovered after 24 h (cfu/tablet).

Antifungal performance and durability tests were conducted according to HG/T 3950-2007. The tested strains included Aspergillus niger, Aspergillus terreus, Paecilomyces varioti, Penicillium funicolosum, Aureobasium pullulans, and Chaetoomium globsum. The spores of the above strains were mixed in equal amounts and set aside. Before use, a negative control test was carried out by spreading the negative control sample (sterile filter paper) on the plate medium and spraying the suspension with a sprayer containing the newly prepared mixed spore suspension to ensure that it was fully and uniformly sprayed on the medium and filter paper. The test was considered invalid and repeated if there was an obvious growth of bacteria on the paper strip after 7d incubation at 28 °C and above 90% RH. After the negative control test was passed, the sample test was conducted. The blank control sample A and antimicrobial-coated sample B were spread on the medium, and the spore suspension was sprayed on the medium and the sample. We conducted 5 parallel tests for each sample. The samples were incubated for 28 d at a temperature of 28 °C and a relative humidity of 90% or more, and the experiment was ended early if the area of mold growth was greater than 10%. The extracted samples were observed immediately. The blank control sample A mold area had to be no less than 10%. Otherwise, it could not be used as a blank control sample for the test. Of the 5 parallel tests for each sample, more than 3 had to show the same long mold level. The sample long mold grade evaluation criteria were as follows:

Level 0: not long, that is, microscopic (magnification 50 times) under the observation of no signs of growth.

Level 1: growth, that is, visible to the naked eye growth, but the growth area covers less than 10%.

Level 2: growth covering an area greater than 10%.

The antiviral performance test was conducted according to ISO-21702. The samples were prepared as 5 × 5 cm squares; a certain amount of virus solution was added dropwise to each test sample and the control sample; and the test solution was gently covered with a 40 mm × 40 mm film and incubated at (25 ± 1) °C, 90% RH for the indicated time (up to 24 h). Then, the surviving virus was eluted with a liquid medium containing the neutralizing agent and recovered virus. The recovered virus was inoculated onto cells in 96-well plates and diluted in a 10-fold gradient with no less than four replicate wells per dilution gradient. The 96-well plates were incubated in a 37 °C, 5% CO_2_ incubator for 3–7 days to record the cytopathic conditions and calculate the rate of virus resistance on the test surface. The TCID_50_ method was used to calculate the virus titer; then, the antiviral activity value and antiviral rate of the specimen were determined. Guangzhou Microbiological Analysis and Testing Center provided the experimental data.

## 4. Conclusions

Methacryloyloxydimethylbenzylammonium bromide (QAS-BN), an antimicrobial monomer with unsaturated double bonds, was synthesized by the Hoffman alkylation reaction. One-step emulsion polymerization was used to prepare the self-stratification core-shell latex particles containing QAS-BN. ^1^H NMR and FTIR spectroscopy results demonstrated that QAS-BN was successfully prepared and could be introduced into the copolymer molecular chain by emulsion polymerization. The introduction of QAS-BN (≤7 wt.%) increased the emulsions’ stability according to the zeta potential results and the calcium ion stability test. When the content of QAS-BN exceeded 3 wt.%, the core-shell structure of the latex particles was observed clearly under TEM. The DSC and DMA results matched the two Tg values obtained due to phase separation, confirming the spontaneous formation of soft-core and hard-shell latex particles in the emulsion reaction. The XPS and SEM results also corroborated the core-shell structure of the latex particles. The relationship between the formation of the core-shell structure and the content of QAS-BN was proved. This core-shell structure with assembled antimicrobial groups provided the coating with better antimicrobial properties. The introduction of the p-CMS/EDA crosslinking system effectively improved the water resistance of the latex film. The antimicrobial coating (QAS-BN content, 2.8 wt.%) prepared by E-7B2p emulsion showed perfect resistance against bacteria, fungi, and viruses. Compared with the mainstream long-chain QAS, the QAS with benzyl groups could achieve the extensive inactivation of envelope-free viruses. This coating inactivated 97.9% of the EV71 virus after 24 h. This study provides a novel idea for the morphological design of latex particles. A new architectural coating with broad-spectrum antimicrobial properties was obtained, with varied applications in public health and safety.

## Figures and Tables

**Table 1:** Effect of QAS-BN dosage on emulsion performance.

Sample	Monomer Conversion (%)	Gelation Rate (wt.%)	Particle Size (nm)	PDI	Zeta Potential (mv)	Stability of Calcium Ions
E-0B0p	99.93	/	82.61	0.017	+36.3	1 wt.%
E-1B0p	99.98	/	82.97	0.033	+45.8	4 wt.%
E-3B0p	99.88	/	83.95	0.040	+46.9	4 wt.%
E-5B0p	98.60	/	88.06	0.066	+48.1	5 wt.%
E-7B0p	96.45	1.21	111.2	0.118	+49.8	5 wt.%
E-9B0p	91.63	5.32	115.4	0.171	+45.8	4 wt.%

**Table 2:** TGA results of poly-acrylate resin with various QAS-BN contents.

Sample	QAS-BN (wt.%)	Core (nm)	Shell (nm)	Size (nm)	Shell (*v*/*v*.%)
E-0B0p	0	87.44	\	87.44	\
E-1B0p	1	87.35	\	87.35	\
E-3B0p	3	87.28	\	87.28	\
E-5B0p	5	51.81	19.09	89.99	66.85
E-7B0p	7	58.44	20.89	100.22	66.00
E-9B0p	9	61.69	21.75	105.20	65.62

**Table 3:** TGA results of latex films with different QAS-BN dosages.

Sample	T_5%_ (°C)	T_10%_ (°C)	T_max_ (°C)
E-0B0p	335.24	352.78	399.84
E-1B0p	353.03	363.37	399.37
E-3B0p	347.73	361.35	394.56
E-5B0p	331.11	360.39	398.14
E-7B0p	305.64	348.13	395.39
E-9B0p	262.54	329.51	394.37

T_5%_ and T_10%_ are the temperatures when the thermal weight loss was 5% and 10%, respectively, and T_max_ is the temperature when the thermal weight loss rate was maximum.

**Table 4:** Effect of p-CMS/EDA content on CACS emulsion performance.

Sample	Conversion Rate /%	Size /nm	PDI	Crosslinkage /%	Water Absorption /%	Water Leaching Rate /%
E-7B0p	96.45	111.2	0.118	21.36 ± 0.61	21.31 ± 0.63	11.23 ± 0.10
E-7B1p	97.98	120.5	0.122	85.06 ± 1.61	11.29 ± 0.50	3.75 ± 0.06
E-7B2p	99.13	124.3	0.134	91.11 ± 1.10	9.99 ± 0.43	4.00 ± 0.03
E-7B3p	99.05	126.5	0.144	91.76 ± 0.72	8.31 ± 0.40	3.66 ± 0.04

**Table 5:** Effect of p-CMS/EDA dosage on CACS emulsion performance.

Sample	Tg-Core/°C	Tg-Shell/°C	E/MPa	Ve/(mol/m^3^)
E-7B1p	42.43	124.59	1.58902	800.88
E-7B2p	41.36	114.48	2.81856	1468.43
E-7B3p	48.96	111.63	2.89276	1496.01

**Table 6:** Result of antimicrobial testing of coating.

Durability Treatment	Testing Microorganism	The Average Number of Recovered Colonies after 24 h (cfu/Piece)		Antibacterial Rate (%)
		Blank Control Sample	Antibacterial Coating Sample	
Before	*E. coli* (AS1.90)	1.0 × 10^7^	<20	>99.99
	*S. aureus* (AS1.89)	2.9 × 10^6^	<20	>99.99
After	*E. coli* (AS1.90)	1.0 × 10^7^	<20	>99.99
	*S. aureus* (AS1.89)	2.9 × 10^6^	<20	>99.99

**Table 7:** The synthetic formulations of emulsions with different contents of QAS-BN.

Sample	Reaction Monomers					Initiator	Emulsifier		Solvent
	MMA (g)	BA (g)	QAS-BN (g)	p-CMS (g)	VTES (g)	AIBA (g)	CTAB (g)	LCN407 (g)	H_2_O (g)
E-0B0p	104.63	104.63	0.00	0	15.75	0.45	2.8	4	275
E-1B0p	103.50	103.50	2.25	0					
E-3B0p	101.25	101.25	6.75	0					
E-5B0p	99.00	99.00	11.25	0					
E-7B0p	96.75	96.75	15.75	0					
E-9B0p	94.50	94.50	20.25	0					
E-7B1p	95.63	95.63	15.75	2.25					
E-7B2p	94.50	94.50	15.75	4.50					
E-7B3p	93.38	93.38	15.75	6.75					
E-7B4p	92.25	92.25	15.75	9.00					

Mass ratio of QAS-BN or p-CMS to total monomer mass. The number in the sample name refers to the content of QAS-BN and p-CMS; for example, E-7B1p indicates that 7 wt.% and 1 wt.% of the total monomer content of QAS-BN and p-CMS, respectively, were involved in the copolymerization reaction.

**Table 8:** Formulation of colorant preparation.

Material	Model	Weight/g
water	/	274.82
emulsifier	LCN407	1.64
dispersant	6208	18.02
defoamer	6393	1.31
antifreeze	propylene glycol	19.66
titanium dioxide	R-706	221.20
800-mesh dicalcium	/	294.94
water-washed kaolin	/	90.12
calcined kaolin	/	147.47

**Table 9:** Formulation of coating preparation.

Material	Model	Weight/g
CACS emulsion	/	100
film-forming additives	DPMA	1
defoamer	2410	0.5
dispersion	/	20–100
leveling agent	BYK-333	0.6
thickening agent	RM-8W	appropriate content

## Data Availability

The data presented in this study supporting the results are available in the main text. Additional data are available upon reasonable request from the corresponding author.

## Supplementary Materials

The following supporting information can be downloaded at: https://www.mdpi.com/article/10.3390/molecules28062795/s1, Scheme S1: Reaction equation for the synthesis of C4, Scheme S2: Reaction equation for the synthesis of C12; Figure S1: ^1^H NMR of C4 monomer, Figure S2: ^1^H NMR of C12 monomer; Table S1: The synthetic formulations of emulsions with different contents of QAS-BN.

## Sample Availability

Samples of the compounds are not available from the authors.

## References

[1] Saud Z., Richards C.A.J., Williams G., Stanton R.J. (2022). Anti-Viral Organic Coatings for High Touch Surfaces Based on Smart-Release, Cu2+ Containing Pigments. Prog. Org. Coat..

[2] Imani S.M., Ladouceur L., Marshall T., Maclachlan R., Soleymani L., Didar T.F. (2020). Antimicrobial Nanomaterials and Coatings: Current Mechanisms and Future Perspectives to Control the Spread of Viruses Including SARS-CoV-2. ACS Nano.

[3] Zhang Z., Whitten D.G., Kell A. (2022). Fluorescent Cellulose Wipe as a New and Sustainable Light-Activated Antibacterial and Antiviral Agent. ACS Mater. Lett..

[4] Kalli S., Araya-Cloutier C., Hageman J., Vincken J.-P. (2021). Insights into the Molecular Properties Underlying Antibacterial Activity of Prenylated (Iso)Flavonoids against MRSA. Sci. Rep..

[5] Zhou Z., Li B., Liu X., Li Z., Zhu S., Liang Y., Cui Z., Wu S. (2021). Recent Progress in Photocatalytic Antibacterial. ACS Appl. Bio Mater..

[6] Xu Z., Zhang C., Wang X., Liu D. (2021). Release Strategies of Silver Ions from Materials for Bacterial Killing. ACS Appl. Bio Mater..

[7] Llorente O., Barquero A., Paulis M., Leiza J.R. (2022). Challenges to Incorporate High Contents of Bio-Based Isobornyl Methacrylate (IBOMA) into Waterborne Coatings. Prog. Org. Coat..

[8] Gao D., Li Y., Lyu B., Jin D., Ma J. (2020). Silicone Quaternary Ammonium Salt Based Nanocomposite: A Long-Acting Antibacterial Cotton Fabric Finishing Agent with Good Softness and Air Permeability. Cellulose.

[9] Wang L.-S., Xu S., Gopal S., Kim E., Kim D., Brier M., Solanki K., Dordick J.S. (2021). Facile Fabrication of Antibacterial and Antiviral Perhydrolase-Polydopamine Composite Coatings. Sci. Rep..

[10] Zhang Y., Chen L., Lin Z., Ding L., Zhang X., Dai R., Yan Q., Wang X. (2019). Highly Sensitive Dissolved Oxygen Sensor with a Sustainable Antifouling, Antiabrasion, and Self-Cleaning Superhydrophobic Surface. ACS Omega.

[11] Sun J., Zhang B., Yu B., Ma B., Hu C., Ulbricht M., Qu J. (2023). Maintaining Antibacterial Activity against Biofouling Using a Quaternary Ammonium Membrane Coupling with Electrorepulsion. Environ. Sci. Technol..

[12] An L., Heo J.W., Chen J., Kim Y.S. (2022). Water-Soluble Lignin Quaternary Ammonium Salt for Electrospun Morphology-Controllable Antibacterial Polyvinyl Alcohol/ Lignin Quaternary Ammonium Salt Nanofibers. J. Clean. Prod..

[13] Zhou Y., Jiang Y., Zhang Y., Tan L. (2022). Improvement of Antibacterial and Antifouling Properties of a Cellulose Acetate Membrane by Surface Grafting Quaternary Ammonium Salt. ACS Appl. Mater. Interfaces.

[14] Daood U., Matinlinna J.P., Pichika M.R., Mak K.-K., Nagendrababu V., Fawzy A.S. (2020). A Quaternary Ammonium Silane Antimicrobial Triggers Bacterial Membrane and Biofilm Destruction. Sci. Rep..

[15] Wen J., Sun Z., Xiang J., Fan H., Chen Y., Yan J. (2019). Preparation and Characteristics of Waterborne Polyurethane with Various Lengths of Fluorinated Side Chains. Appl. Surf. Sci..

[16] Xu Q., Ma J., Zhou J., Wang Y., Zhang J. (2013). Bio-Based Core-Shell Casein-Based Silica Nano-Composite Latex by Double-in Situ Polymerization: Synthesis, Characterization and Mechanism. Chem. Eng. J..

[17] Zhi L., Shi X., Zhang E., Gao C., Gai H., Wang H., Liu Z., Zhang T. (2022). Synthesis and Performance of Double-Chain Quaternary Ammonium Salt Glucosamide Surfactants. Molecules.

[18] Zeng M., Xu J., Luo Q., Hou C., Qiao S., Fu S., Fan X., Liu J. (2020). Constructing Antibacterial Polymer Nanocapsules Based on Pyridine Quaternary Ammonium Salt. Mater. Sci. Eng. C.

[19] Kawabata N., Yamazaki K., Otake T., Oishi I., Minekawa Y. (1990). Removal of Pathogenic Human Viruses by Insoluble Pyridinium-Type Resin. Epidemiol. Infect..

[20] Wood A., Payne D. (1998). The Action of Three Antiseptics/Disinfectants against Enveloped and Non-Enveloped Viruses. J. Hosp. Infect..

[21] Sundberg D. (2020). Structured, Composite Nanoparticles from Emulsion Polymerization—Morphological Possibilities. Biomacromolecules.

[22] Sahu A., Kumar D. (2022). Core-Shell Quantum Dots: A Review on Classification, Materials, Application, and Theoretical Modeling. J. Alloys Compd..

[23] Zou H., Luo Z., Yang X., Xie Q., Zhou Y. (2022). Toward Emerging Applications Using Core-Shell Nanostructured Materials: A Review. J. Mater. Sci..

[24] Duan S., Wu R., Xiong Y.-H., Ren H.-M., Lei C., Zhao Y.-Q., Zhang X.-Y., Xu F.-J. (2022). Multifunctional Antimicrobial Materials: From Rational Design to Biomedical Applications. Prog. Mater. Sci..

[25] Wang J., Sun M., Zhang X., Wang F., Song J., Zhang W., Wang Z., Li M. (2022). Nonmetal (S or Se)-Bridged Core Shell Electrodes for Promoting Electrochemical Performance. Adv. Energy Mater..

[26] Yang J., Mu Y., Li X. (2022). Morphology of Multilayer Core-Shell Emulsion: Influence of Crosslinking Agent. Mater. Lett..

[27] Cheaburu-Yilmaz C.N., Ozkan C.K., Yilmaz O. (2022). Synthesis and Application of Reactive Acrylic Latexes: Effect of Particle Morphology. Polymers.

[28] Zeng Y., Yang W., Xu P., Cai X., Dong W., Chen M., Du M., Liu T., Jan Lemstra P., Ma P. (2022). The Bonding Strength, Water Resistance and Flame Retardancy of Soy Protein-Based Adhesive by Incorporating Tailor-Made Core-Shell Nanohybrid Compounds. Chem. Eng. J..

[29] Chen D., Ding M., Huang Z., Wang Y. (2021). Styrene–Acrylic Emulsion with “Transition Layer” for Damping Coating: Synthesis and Characterization. Polymers.

[30] Mu Y., Han L., Du D., Yang Y., Li X. (2022). Reversible Thermochromic and Thermal Insulation Performances of K2O·nSiO2 Based Fire-Resistant Glass via in-Situ Preparation. Constr. Build. Mater..

[31] Ma J., Liu Y., Bao Y., Liu J., Zhang J. (2013). Research Advances in Polymer Emulsion Based on “Core-Shell” Structure Particle Design. Adv. Colloid Interface Sci..

[32] Mu Y., Yang Y., Xu L., Zhang Y., Hu Y., Xu Z. (2021). In-Situ Preparation and Performance of Cold Resistant K2O·5SiO2 Based Anti-Fire Glass. Constr. Build. Mater..

[33] Hou Y.-K., Zhang Z.-J., Li R.-T., Peng J., Chen S.-Y., Yue Y.-R., Zhang W.-H., Sun B., Chen J.-X., Zhou Q. (2023). Remodeling the Tumor Microenvironment with Core-Shell Nanosensitizer Featuring Dual-Modal Imaging and Multimodal Therapy for Breast Cancer. ACS Appl. Mater. Interfaces.

[34] Yao M., Shi X., Zuo C., Ma M., Zhang L., Zhang H., Li X., Yang G.-Y., Tang Y., Wu R. (2020). Engineering of SPECT/Photoacoustic Imaging/Antioxidative Stress Triple-Function Nanoprobe for Advanced Mesenchymal Stem Cell Therapy of Cerebral Ischemia. ACS Appl. Mater. Interfaces.

[35] Zhou X., Li P., Wu X., Lin X., Zhao L., Huang H., Wu J., Cai H., Xu M., Zhou H. (2022). Multifunctional Biosensor Constructed by Ag-Coating Magnetic-Assisted Unique Urchin Core Porous Shell Structure for Dual SERS Enhancement, Enrichment, and Quantitative Detection of Multi-Components Inflammatory Markers. Biosens. Bioelectron..

[36] Wu P., Cui Y., Sun Z., Cao M., Liu Y., Fu K., Zhou Y. (2023). Sodium Alginate Coupled with Organosilane Quaternary Ammonium Salt for the Antibacterial Application. Cellulose.

[37] Zhang X., Sun J., Chen Y., Chen Q., Bai L., Gu J., Li Z. (2022). Engineered Latex Particles Using Core-Shell Emulsion Polymerization: From a Strawberry-like Surface Pattern to a Shape-Memory Film. ACS Appl. Polym. Mater..

[38] Mu Y., Qiu T., Li X., Guan Y., Zhang S., Li X. (2011). Layer-by-Layer Synthesis of Multilayer Core−Shell Latex and the Film Formation Properties. Langmuir.

[39] Lingxiao W., Guilong X., Min T., Yun L. (2023). Preparation and Properties of Quaternary Phosphonium Salt Containing Poly-Acrylate Emulsion. Prog. Org. Coat..

[40] Wu C., Dai D., Zhao X., Wang H., Li T. (2022). One-Step Rapid Fabrication of MOF@polymer Core-Shell Particles through Non-Solvent Induced Surface Deposition. J. Mater. Chem. A.

[41] Bilgin S., Tomovska R., Asua J.M. (2016). Fundamentals of Chemical Incorporation of Ionic Monomers onto Polymer Colloids: Paving the Way for Surfactant-Free Waterborne Dispersions. RSC Adv..

[42] Buback M., Hutchinson R.A., Lacík I. (2023). Radical Polymerization Kinetics of Water-Soluble Monomers. Prog. Polym. Sci..

[43] Shen J., Lin X., Liu J., Li X. (2019). Effects of Cross-Link Density and Distribution on Static and Dynamic Properties of Chemically Cross-Linked Polymers. Macromolecules.

[44] Zhao S., Abu-Omar M.M. (2016). Renewable Epoxy Networks Derived from Lignin-Based Monomers: Effect of Cross-Linking Density. ACS Sustain. Chem. Eng..

[45] Xie H., Zhang S., Duan H. (2004). An Ionic Liquid Based on a Cyclic Guanidinium Cation Is an Efficient Medium for the Selective Oxidation of Benzyl Alcohols. Tetrahedron Lett..

[46] Meek K.M., Elabd Y.A. (2015). Alkaline Chemical Stability of Polymerized Ionic Liquids with Various Cations. Macromolecules.

[47] Strehmel V. (2015). Application of Ionic Liquids in Synthesis of Polymeric Binders for Coatings. Prog. Org. Coat..

