# Supplementary material for: One-Step Synthesis of Self-Stratification Core-Shell Latex for Antimicrobial Coating
Source: Molecules. 2023 Mar 20;28(6):2795. doi: 10.3390/molecules28062795 (PMC10052133; doi:10.3390/molecules28062795)
Supplement: Supplementary file 1 [file molecules-28-02795-s001.zip › molecules-2271000-supplementary.pdf]

Supplementary Materials

One-Step Synthesis of Self-Stratification Core–Shell Latex for Antimicrobial Coating

Guanzhou Zhen [1], Yuanchun Mu [1,2,*], Peichen Yuan [1], Yankun Li [1] and Xiaoyu Li [1,2,*]

[1] Beijing Engineering Research Center of Synthesis and Application of Waterborne Polymer, Beijing University of Chemical Technology, Beijing 100029, China
[2] State Key Laboratory of Organic-Inorganic Composite, Beijing University of Chemical Technology, Beijing 100029, China
* Correspondence: 2021500068@mail.buct.edu.cn (Y.M.); lixy@mail.buct.edu.cn (X.L.)

S1. Synthesis of QAS-BN homopolymer P(QAS-BN)

20. g of QAS-BN was dissolved in 80g of deionized water, added to a four-necked flask with nitrogen gas and heated to 87.5°C. Add 2 g of initiator AIBA and react under stirring condition for 4 h. The aqueous solution of P-QAS-BN was obtained. The aqueous solution of P(QAS-BN) was added to acetonitrile in the ratio of 1:10 and washed 3 times, filtered, and dried to obtain a white solid powder.

S2. Control sample formulation in antiviral testing

S2.1. Anti-microbial monomer synthesis method

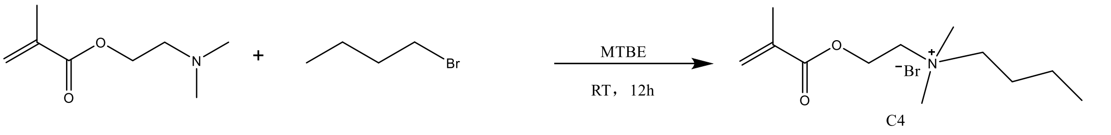

DMAEMA 1-bromobutane *N*-(2-(methacryloyloxy)ethyl)-*N*,*N*-dimethylbutan-1-aminium

Scheme S1. Reaction equation for the synthesis of C4.

C4 was accomplished by the Hoffman alkylation reaction of 1-bromobutane with DMAEMA. The reaction equation is given in **Scheme S1**. Specifically, 1-bromobutane and DMAEMA were mixed in hexane in a 1:1 ratio and stirred to react fully, and the solid pre-product was obtained by filtration. Next, the product was washed using hexane and acetone and dried under a vacuum to get a solid white product. The ^{1}H NMR and IR spectra of the monomer are given in **Figure S1**.

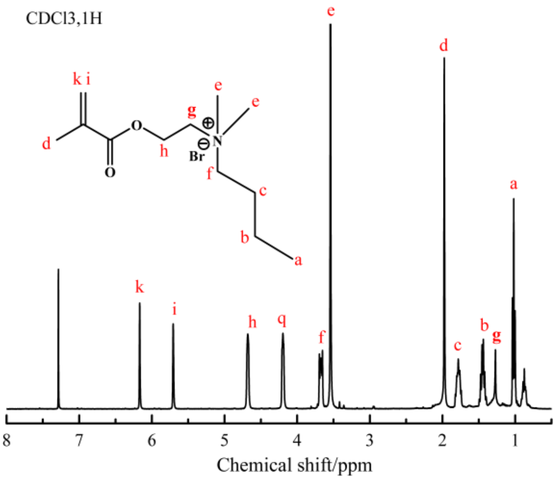

Figure S1. ¹H NMR of C4 monomer.

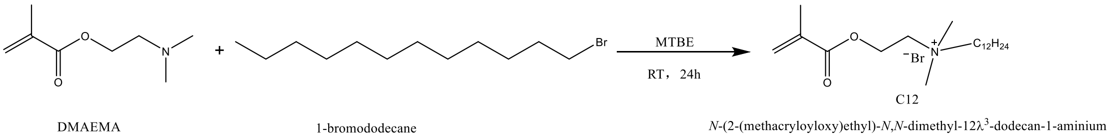

Scheme S2. Reaction equation for the synthesis of C12.

C12 was accomplished by the Hoffman alkylation reaction of Bromododecane with DMAEMA. The reaction equation is given in **Scheme S1**. Specifically, Bromododecane and DMAEMA were mixed in hexane in a 1:1 ratio and stirred to react fully, and the solid pre-product was obtained by filtration. Next, the product was washed using hexane and acetone and dried under a vacuum to get a solid white product. The ¹H NMR and IR spectra of the monomer are given in **Figure S2**.

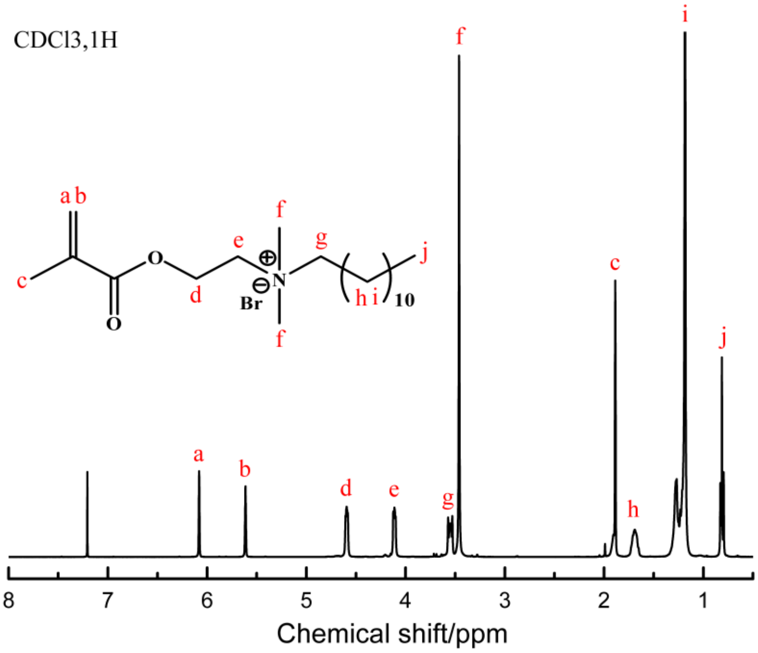

Figure S2. ¹H NMR of C12 monomer.

S2.2. Synthetic formulation of emulsion for the control sample

Table S1. The synthetic formulations of emulsions with different contents of QAS-BN.

Sample	Reaction monomers MMA (g)	BA (g)	C12 (g)	C12 (g)	p-CMS (g)	VTES (g)	initiator AIBA (g)	Emulsifier CTAB (g)	LCN407 (g)	Solvent H_2O (g)
CONTROL	94.50	94.50	13.5	6.75	4.50	15.75	0.45	2.8	4	275

*Mass ratio of QAS-BN or p-CMS to total monomer mass; *The number in the sample name refers to the content of QAS-BN and p-CMS, for example.E-7B1p indicates that 7 wt.% and 1 wt.% of the total monomer content of QAS-BN and p-CMS are involved in the copolymerization reaction. .
